# Proportionate and disproportionate reporting of different infectious complications of disease modifying therapy in multiple sclerosis

**DOI:** 10.1186/s42466-026-00544-x

**Published:** 2026-10-21

**Authors:** Frederike Kadgien, Katja Hummel, Peter Trillenberg

**Affiliations:** 1 Infectious Disease Clinic, University Hospital of Schleswig-Holstein, Campus Lübeck, Ratzeburger Allee 160, Lübeck, Germany; 2 Department of Neurology, University Hospital of Schleswig-Holstein, Campus Luebeck, Ratzeburger Allee 160, 23538 Luebeck, Germany

**Keywords:** Multiple Sclerosis, Disease modifying therapy, Progressive multifocal Leukoencephalopathy

## Abstract

**Introduction:**

Progessive multifocal leukoencephalopathy (PML) is a complication of immunoincompetence due to disease or immunosuppressive therapy and in particular due to disease modifying therapy (DMT) in multiple sclerosis. It is not clear however, whether there is a specific vulnerability to PML or whether this is due to immunosuppression per se. The same holds for the combination of cryptococcal disease and Fingolimod.

**Methods:**

We used the reporting odds ratio (ROR) as calculated from entries to Vigibase for a sample of drugs used for DMT (Alemtuzumab, Cladribine, Fingolimod, Dimethyl fumarate, Glatirameracetat, Interferon beta-1a, Interferon beta-1b, Natalizumab, Ocrelizumab, Ofatumumab, Ozanimod, Peginterferon-beta-1a, Ponesimod, Siponimod, and Teriflunomide) and infections with the varicella zoster virus (VZV), PML, and cryptococcal and pneumococcal disease as a surrogate for the frequency of these events. Principal component analysis was used to identify outliers of the general relation between the RORs for the 4 infections studied. A plot of log(ROR) for the other infections as a function of log(ROR) of VZV infections was used to characterize the outliers more clearly.

**Results:**

The associations of PML/Natalizumab and Cryptococcal disease/Fingolimod were reported significantly more frequently than expected from the general reporting pattern as extracted from the PCA. They were the only combinations which were outside the confidence intervals of the otherwise linear correlations of log(ROR) with log(ROR) for VZV.

**Conclusion:**

These associations are candidates for which specific effects of Natalizumab and Fingolimod should be identified that increase vulnerability to respective infections.

**Supplementary Information:**

The online version contains supplementary material available at https://doi.org/10.1186/s42466-026-00544-x.

## Introduction

Multiple Sclerosis (MS) is a chronic autoimmune disease of the central nervous system. Anti-inflammatory drug therapy to reduce disease activity is termed “disease modifying therapy” (DMT) and covers a spectrum from immunomodulation to immunosuppression which may predispose to infectious complications [1]. As an example progressive multifocal leukoencephalopathy (PML) caused by the JC virus, which is extremely rare in immunocompetent hosts, occurs in patients treated with Natalizumab [2]. However, there have also been reports of PML in association with Dimethyl fumarate [3] and with Fingolimod [4–6]. Therefore, a significantly increased PML risk associated with a given drug (as reflected in a signal in a pharmacovigilance analysis) could reflect a specific vulnerability to the JC Virus or a general vulnerability to otherwise rare infectious agents. As another example, both infections with Cryptococcus neoformans [7, 8] and with the varicella zoster virus (VZV) [9, 10] have been highlighted as a complication of therapy with Fingolimod. For VZV/Fingolimod there are comparisons with other DMTs [10] (indicating at least a more frequent reporting than for other DMT) but there are no simultaneous comparisons of different diseases and drugs that might reveal candidates for specific associations.

We therefore compared various immunosuppressive drugs used in MS therapy with respect to infections VZV, the JC virus, pneumococci and cryptococci. As a quantitative surrogate of the risk of infection we used the reporting odds ratio (ROR) in a pharmacovigilance data base. We hypothesized that correlations between the risks for different infectious complications would reflect a common immunosuppressive effect of the various drugs and that deviations from these correlations would reflect specific vulnerabilities to single infectious agents.

## Methods

For a predefined list of 15 drugs (Alemtuzumab, Cladribine, Fingolimod, Dimethyl fumarate, Glatirameracetat, Interferon (IFN) beta-1a, IFN beta-1b, Natalizumab, Ocrelizumab, Ofatumumab, Ozanimod, Peginterferon-beta-1a, Ponesimod, Siponimod, Teriflunomide) we retrieved reports to VigiBase [11] via vigiaccess (access date: Feb. 26th, 2024). In the output, we searched for entries related to the VZV, to the JC Virus, to pneumococci and to cryptococci. For each combination of drug and infectious agent we calculated the ROR according to standard definition [12].

With the 15 × 4 matrix of the RORs of 15 drugs and 4 infectious complications we performed a principal component analysis (PCA [13–15]). The calculations were carried out with the function “PCA” of Matlab (MATLAB and Statistics Toolbox Release 2021a, The MathWorks, Inc., Natick, Massachusetts, United States). The PCA (with the matrix dimensions of our data set) successively determines as new coordinates linear combinations (principal components, PC) of the original 4 RORs that explain the maximum of the remaining variance after the previous step. It turned out that the first 2 components explained most of the variance. These were retained for the remainder of the analysis.

The PCA result is presented in a “biplot” with the first 2 new coordinates PC1 and PC2. The 4 arrows in the biplot show the directions in which the new coordinates increase with increasing ROR for one particular infection. The 15 points in our biplot show the new coordinates of the original 15 data points. To detect a significant distance from the bulk of data points we used Hotellings T² statistic. The critical value for T² was calculated as (k * (*n* − 1) / (n - k)) * F_krit_(1 - alpha, k, n - k) with *n* = 15, k = 2, alpha = 0.05. The calculation was repeated with exclusion of each of the non-significant drugs to confirm the result.

To further illustrate the results, we then performed linear regressions with the log of the VZV related ROR as independent variable and the log of the other RORs as dependent variable. We calculated the upper 95% percentile as a function of the independent variable. Combinations infection vs. drug with no reported cases were excluded from the analysis. This part of the analysis was carried out with Matlab functions “fitlm” and “predict” with prediction option „observation“.

## Results

A total of 1,017,231 reports were filed for the drugs under study, 8638 of which referred to infections with the VZV, 2617 to PML, 167 to cryptococcal disease, and 62 to pneumococcal disease. Raw figures (number of reports for each combination of drug and infectious complication) are given in the supplement. The table also summarizes RORs and their confidence intervals for a given complication.

The RORs for Glatirameracetat were the lowest among all drugs for VZV, cryptococci and pneumococci, and third lowest for PML. There were also low RORs < 1 for the interferons, reflecting a reporting frequency below average in the group of drug studied.

RORs for Fingolimod were high for all four infections (top rank for cryptococci, rank 2 for Zoster, rank 3 for PML and rank 4 for pneumococci), but even in that context cryptococci scored very high (ROR 25,6 for Cryptococcus, as compared to 3.1, 2.2 and 1.6 for Zoster, Pneumococcus and PML, respectively).

**Fig. 1:**
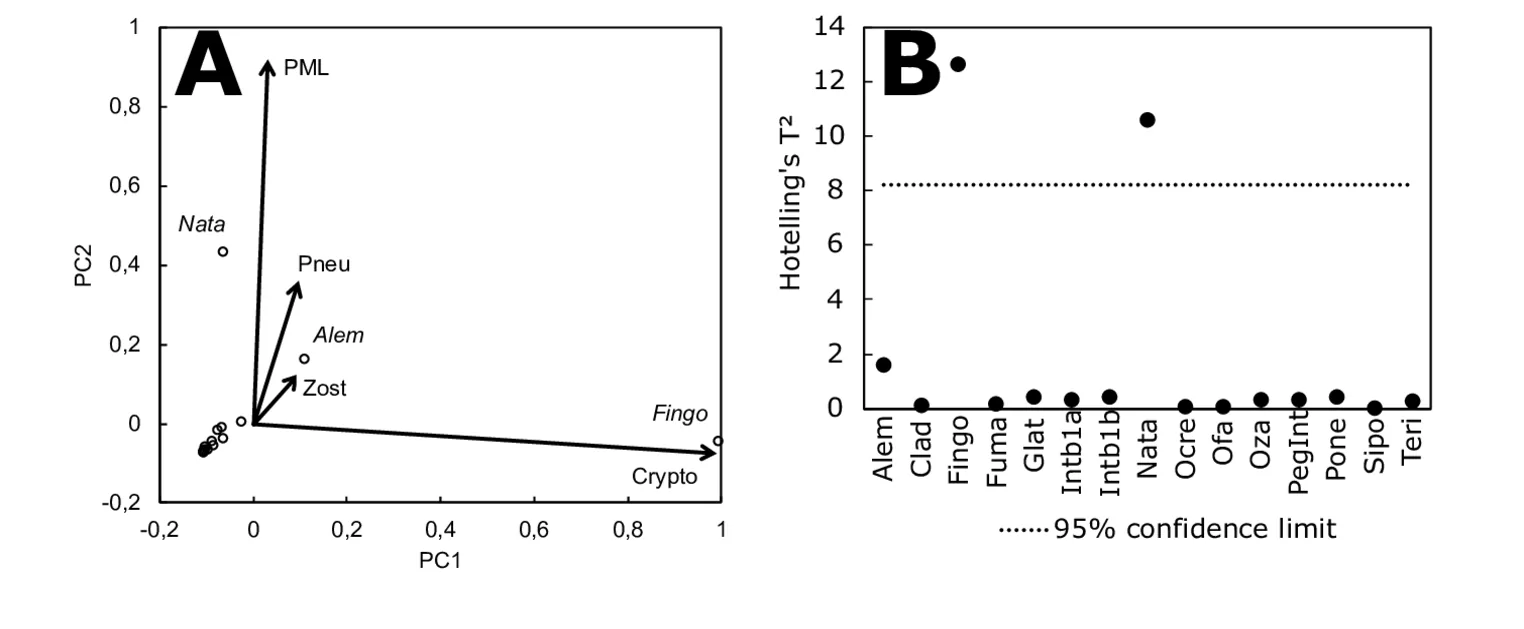
Results of Principal component analysis. A: Biplot with principal components PC1 and PC2. Arrows indicate the variation of PC1 And PC2 with the original parameter ROR for the four infections studied (indicated by “PML”, “Pneu”, “Zost”, “Crypto”). Circles indicate the drugs, with outliers indicated by “Nata”, “Alem“, “Fingo”. B: Hotelling’s T² for the 15 drugs under study in the PCA with 2 components retained. The 95% confidence limit indicates that Fingolimod (“Fingo”) and Natalizumab (“Nata”) significantly deviate from the overall pattern

In the PCA the first two components explained 87% of the total variance. The biplot with PC1 and PC2 (Fig. 1A) revealed that most of the data points clustered along the diagonal close to the origin. Fingolimod, Natalizumab and Alemtuzumab were separated from this cluster with decreasing distance, thus making these three drugs candidates for a significant result. The arrows representing the variation of the new coordinates with the original RORs showed that the data points scattered parallel to the variation with the zoster ROR. Hotelling’s T² was significant for Fingolimod (12.67, T²_crit_=8.2) and for Natalizumab (T²=10.6) but not for alemtuzumab (T²=1.64, Fig. 1B). The deviation of the Fingolimod data point occurred in the direction of the vector reflecting variations of the Cryptococcal infection ROR. A similar result was obtained for the combination Natalizumab/PML. Fingolimod and Natalizumab remained significant if any of the non-significant drugs was excluded from the analysis.

**Fig. 2:**
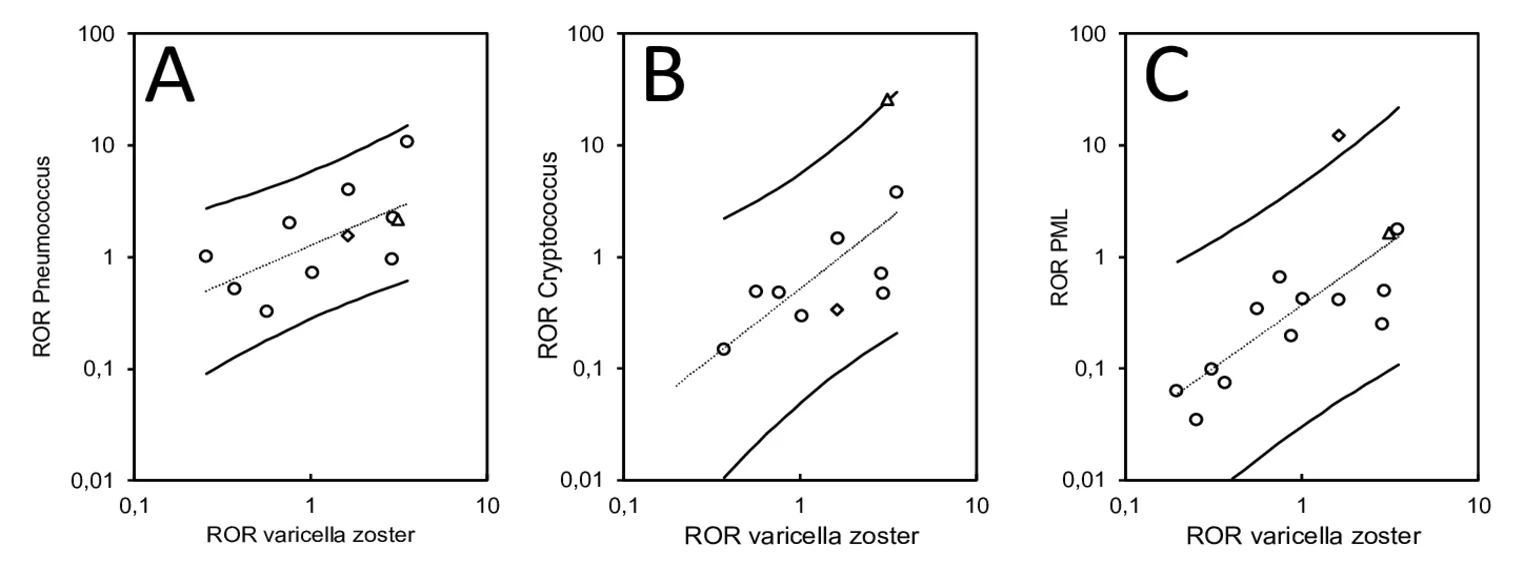
Log-log plot of RORs for pneumococcal disease (**A**), cryptococcal disease (**B**) and PML (**C**) vs. ROR of VZV related disease, with 90% confidence interval. Thus, the upper limb of the confidence interval represents the 95% percentile. Diamond: Natalizumab, triangle: Fingolimod, all other drugs are coded as circles

For all infections under consideration log ROR increased linearly with log ROR for VZV related infections (slope of regression line in Fig. 2 significantly > 0). The only RORs that were above the 95% percentile of the regressions were cryptococcal infections for Fingolimod (ROR 25.6, CI 18.3–35.8, 95% percentile of linear fit 24.7, Fig. 2B), and PML in the case of Natalizumab (ROR 12.2, CI 11.2–13.2, 95% percentile 7.9, Fig. 2C), confirming the impression from the biplot of the PCA.

## Discussion

### Is there a general pattern of reporting of infectious complications characteristic for a given drug?

In our PCA, the scattering of most of the data occurred close to the direction corresponding to a variation of the VZV infection related ROR. Also, we expected reliable counts of reports from a frequent disease (8600 reports for VZV, as compared to 167 for cryptococcal disease). On the other hand, we later discuss many detrimental influences on the data underlying our study. Therefore, we first compare our VZV ROR results with probably more reliable incidence rates from the literature.

Whereas in our analysis the ROR for VZV/Fingolimod is 10 times higher than the ROR VZV/IFN beta, an essentially identical incidence rate (as inferred from a health care provider’s database) for herpes zoster was found for IFN on one hand, and Fingolimod, Natalizumab and Rituximab on the other hand [16]. With IFN as a reference the largest risk ratio among these three drugs was 1.77 for Herpes zoster and Fingolimod which just missed significance. The incidence rate with IFN was, however, surprisingly high in that study. Alemtuzumab, Cladribine and Ocrelizumab, which all yielded high RORs in our study, were not included in that study. This also might in part explain the discrepancy. Another analysis of VZV related infections reports much lower incidence rates of 250/100,000 patient-years (pt-yr) for IFN and 500/100,000 pt-yr for placebo, in contrast to high incidence rates > 1000/100,000 pt-ys for Natalizumab, Fingolimod and Rituximab and > 1500/100,000 pt-ys for Alemtuzumab [10]. The baseline rate in that study is similar to studies unrelated to MS [17]. This is in accordance with the clinical impression that interferons do not cause infectious complications [18]. On the other hand, our RORs for IFNs/VZV infections were significantly below 1 indicating a reduced risk (as compared to a class of drugs with a probably increased risk). This suggests that an ROR of 1 (representing the average of the drugs included in our study) already implies an increased risk for the infections addressed here.

As compared to the high VZV incidence rates for Natalizumab, Fingolimod, and Alemtuzumab (all > 1000/100000 pt-ys as mentioned above [10]), a pooled analysis of the control groups in clinical trials with Cladribine showed a much lower incidence rate of 198/100,000 pt-ys [19]. More consistent with the high ROR in our data (2.90, CI 2.62 to 3.21), the VZV incidence rate as inferred from the Cladribine groups in CLARITY, CLARITY Extension, ORACLE-MS and the PREMIERE registry was 833/100,000 pt-ys [19]. A similar incidence rate (890/1000000 pt-ys) was estimated in another analysis of the same data set [20]. Similarly, in a Swedish register-based cohort study, the odds ratio for “herpes antiviral use” was 2.1 for both Fingolimod and Natalizumab if IFN and Glatiramer acetate served as a reference [21]. The CLARION study [22] reports incidence rates of Herpes zoster for Cladribine (551/100000 pt-yr) and Fingolimod (327/100000 pt-yr), respectively. The confidence intervals were wide, however, and do not indicate a significant difference, similar to our ROR findings (ROR 2.9 for Cladribine and 3.1 for Fingolimod), although the absolute rates are higher than in the studies mentioned before.

In summary, incidence rates from most but not all sources for incidence rates of VZV infections follow the pattern of RORs as obtained from our data.

With respect to some infectious diseases defined clinically rather than by infectious agent, significantly increased risk ratios have also been demonstrated in the above mentioned study based on insurance data [16], e.g. for urinary tract infections and Rituximab, Natalizumab and Fingolimod. Note, that with the general population as a reference, incidences for all infections addressed (with the exception VZV related diseases) were increased. This difference must reflect an MS related risk (impairment in mobility, impaired bladder control etc.). Accordingly, incidence of many infectious complications was increased even in untreated MS patients. Also note that the comparison with the general population requires careful adjustment of age and gender [21]. This is much less of a problem in comparisons within a group of MS drugs although some of the reports that entered our analysis must originate from patients who received the drugs for diseases other than MS (e.g. Fumarate for psoriasis) and for whom different sociodemographic characteristics might apply.

Another interesting approach to quantify immune(in)competence is to study antibody and T-cell response to vaccination. This has been done for the mRNA vaccination against SARS-CoV-2 in MS patients treated with Ocrelizumab, Fingolimod, Cladribine and IFN [23]. Whereas both the T-cell response and the antibody response was reduced for Fingolimod, only the antibody response was impaired for Ocrelizumab [23, 24]. For IFN and Cladribine there was only a slight reduction in the T-cell response. This is consistent with an ROR below one (small risk) for IFN and Cladribine vs. pneumococcal and cryptococcal disease and PML, but not with an ROR of 2.90 of Cladribine/VZV. Unfortunately, Natalizumab was not included in that study. In another study with the same vaccine both T-cell and antibody response were close to normal for Natalizumab [25]. Such findings point to mechanisms unrelated to host defense as a cause for the high PML risk under therapy with Natalizumab. Interestingly, in that study [25] the impairment of T-cell response and the impairment of antibody response were correlated and did not show an expected selective impairment in antibody response for Ocrelizumab [23].

### Risk for a specific infectious complication characteristic for a given drug

PML and cryptococcal infections under therapy with Natalizumab and Fingolimod, respectively, were reported significantly more often than one would expect based on all other drug/complication combinations. Note, that the RORs for Fingolimod were generally high, but that the combination with Cryptococci was high even in that context. Only a simultaneous analysis of different infections can show that even in the context of frequent reports with Fingolimod in general, the combination Fingolimod/Cryptococci is reported more frequently. Similar to our above discussion of the VZV incidence rates we now compare our RORs with incidence rates available from other sources with respect to these two infections.

#### Comparisons with other sources for frequency of PML and cryptococcal infections

From the Novartis safety database an incidence rate of 8/100,000 pt-yr for cryptococcal meningitis in patients with Fingolimod was estimated [7]. For comparison the annual incidence rate in the general population of the United States was 0.3/100,000 person-years in the HIV negative population and between 1.7 (1997) and 0.8 /100,000 (2009) in HIV positive persons [26].

With respect to PML, the incidence rate was 0.11/100 000 pt-yr in the general population (2011–2013) [27] whereas for patients treated with Natalizumab the incidence was 150–350/100,000 pt-yr in France [28] with some variation from year to year (for comparison in HIV-positive patients: 70/100000 pt-yr [29] ), Our ROR relation of 1:4 (Dimethyl fumarate: Fingolimod) is approximately reproduced in the incidence rates of 1/100,000 patient years for Dimethyl fumarate as estimated from the Biogen data base [30] and 3/100,000 patient years for Fingolimod from the Novartis data base in 2018 [4]. For a cohort of MS patients extracted from the FDA Adverse Event Reporting System (FAERS) with registration between July 2015 and June 2017 odds ratios for the diagnosis of PML have been calculated in a group of drugs partially overlapping with our study [31]. The correlation of log(ROR) from our study and log(OR) from that study was very high (R²=0.96) (overlapping drugs Fingolimod, Dimethyl fumarate, Glatirameracetat, IFN beta, Natalizumab included). The correlation per se is reassuring, and the high degree of correlation is partially explained by the fact that the cases from that study are included in our study as well. Note that conflicting data with a higher risk in Fumarate as compared to Fingolimod in a metaanalysis is due to using prevalences rather than incidence rates [32]. Furthermore, Oshima’s data on Fingolimod enter Rindi’s analysis with a very small weight of 0.1% although that study contributes 16,000 patients as compared to Berger’s sample of 21,000 patients, which is weighted with 99%.

#### Possible mechanisms of specific vulnerability: Natalizumab - PML

Our data provide a signal that there could be specific mechanisms that render patients with Natalizumab therapy particularly vulnerable to PML and patients with Fingolimod particularly vulnerable to cryptococci.

Natalizumab is an antibody to α4 integrin which is expressed on lymphocytes and binds to the vascular cell adhesion molecule-1 (VCAM-1) of the endothelium. In patients undergoing natalizumab treatment migration of lymphocytes to the brain is therefore impaired. JC virus causes asymptomatic infection in immunocompetent individuals and clinical disease, including PML, primarily in immunosuppressed patients [33], with essentially absent pleocytosis [34]. Seroprevalence increases with age with an overall seropositivity of 60% [35, 36].

Are there possible biological mechanisms that could explain our finding that with Natalizumab the reporting frequency of PML, but not of VZV, pneumococcal infection and cryptococcal infection is out of the expected range?

Several effects of Natalizumab on JC virus pathogenesis have been proposed [36]: Natalizumab forces latently infected CD34 + cells from the bone marrow into the circulation, and thus facilitates the spread of the virus. Additionally, Natalizumab upregulates transcription factors SpiB and miRNA126, which enhance JC virus replication. JC virus replicates in oligodendrocytes of the brain and causes their lysis. The control of JC virus infection and reactivation strongly depends on cellular immunity, particularly on the function of JC virus specific cytotoxic CD-8 + and CD-4 + T-cells [37, 38]. In MS patients treated with Natalizumab developing PML JC virus specific T-cells and T-cell responses are absent, comparable to non PML patients [39]. JC virus control can be reinstalled successfully by allogenic transplantation of JC virus specific T-cells [40–42]. In contrast to JC virus, pneumococci and cryptococci disrupt the blood brain barrier as part of their pathogenesis [43], and thus, lymphocytes can enter the brain and meninges to control infection.

#### Possible mechanisms of specific vulnerability: Fingolimod-Cryptococci

Fingolimod is a Sphingosine (SIP) 1-phosphate receptor modulator that keeps lymphocytes from leaving lymph nodes [5]. Thus, Fingolimod reduces peripheral lymphocyte counts, and lymphocytic invasion into the brain [44]. Patients on fingolimod treatment have an increased risk of infections including severe herpes infections, respiratory infections, PML and cryptococcosis [5, 45]. All published cases with cryptococcosis presented with lymphopenia and frequently with CD-4 + T-cell counts less than 100 cells/µL [46]. Treatment duration with Fingolimod > 2 years, age and immunosenescense [47] have been suggested as additional risk factors. Since SIP receptors are expressed on e.g. cardiomyocytes there are noninfectious adverse events in connection with SIP inhibitors.

Interestingly, we were able to detect an out of proportion ROR for cryptococcal infection for Fingolimod only. For the other SIP 1 inhibitors of which Ponesimod and Siponimod were included in this study the number of Cryptococcus associated reports was small (2 for Siponimod and 0 for Ponesimod), probably because of the more recent introduction of these drugs. For such low counts the estimation of the ROR is imprecise. Theoretically, different rates of cryptococcal infections could be possible due to different receptor affinity (Ozanimod and Siponimod: S1P-1, -5; Ponesimod: S1P-1, Fingolimod: S1P-1, -3, -4, -5).

Cryptococci are dimorphic fungi. Most C. neoformans infections occur in immunosuppressed individuals, although genetic predisposition to the disease has been associated with defects in the mannose-binding lectin or polymorphisms in the Fcy receptor 2b. C. gattii infections are rare but affect immunocompetent patients and outbreaks have been described [48]. Manifestations of cryptococcosis in MS patients treated with fingolimod commonly affect the lung and CNS [46]. As a part of their virulence strategy cryptococci secrete cytokines that influence dendritic cells to activate TH2 cells. These TH2 cells produce anti-inflammatory cytokines that hinder elimination of cryptococci by the phagosomes of macrophages [36]. Hindering phagocytosis is a key virulence factor of cryptococci. It is believed that cryptococci use macrophages as Trojan horses to cross the blood brain barrier [49]. An effective immune defense against cryptococci in immunocompetent hosts involves CD-4 + TH1 cells secreting IFN gamma, IL-17 and activating macrophages. TH1 response and high levels of IFN gamma are associated with fungal clearance and TH2 response with poor control of cryptococci [50–52]. Furthermore, Fingolimod may induce an immune phenotype similar to that observed in immunosenescense and early thymic involution in patients enhancing susceptibility to cryptococcal infection [47]. MS patients with severe lymphopenia may be at highest risk of disease similar to HIV patients with advanced disease and lymphopenia. Cryptococcal antigen positivity correlates with disease development [53]. Antigen screening of MS patients with a CD-4 + T-cell count below 100/ µL, as recommend by the European AIDS clinical society for HIV infected patients, may help to identify those at risk and enable a prompt initiation of preemptive therapy with fluconazole (EACS guidelines 2025).

### Limitations

Our study is based on entries in the VigiBase data base. VigiBase receives reports from national drug safety agencies of many different countries with probably very different strategies and options for the treatment of Multiple Sclerosis. Part of the entries in the database stem from spontaneous reports. Spontaneous reporting can be subject to many distorting influences that confound the relation between reporting counts and true incidence rate [54, 55]. A probably more reliable measure are incidence rates as reported in controlled trials with rigorous rules for patient surveillance and reporting of adverse events. In previous sections of the discussion, we tried to compare ratios of RORs with corresponding ratios of incidence rates, but these are available for a very limited number of combinations.

At the same incidence, spontaneous reports might be more likely in severe complications (PML as opposed to VZV related disease). Reports might be less likely for complications that are so far unknown and for which no plausible mechanism is suspected, or for familiar complications with clear mechanism. The uncertainty how closely disproportional reporting reflects true risk is expressed with the term “signal” for a significant result (as opposed to “significantly increased risk”).

With the intense coverage of PML occurrence under Natalizumab the reporting probability is probably increased (“notoriety bias” [54]), . One can assume, however, that this effect generalizes to any case of PML under DMT. Therefore, this bias would shift all points in Fig. 2 up, leaving the relation between drugs unchanged.

Since VigiBase does not provide temporal statistics on single combinations of drug and event we cannot analyze temporal variations in the reporting frequency. Such data could help to assess the biases mentioned above. Furthermore, they could reflect the success of personalized strategies for PML risk control in Natalizumab therapy, based on antibody findings and treatment history [56]. All these factors cannot be differentiated in the data underlying our study.

## Supplementary Information

Supplementary Material 1

## Data Availability

All data supporting the findings of this study are available within the paper and its Supplementary Information.
