# Supplementary material for: Proportionate and disproportionate reporting of different infectious complications of disease modifying therapy in multiple sclerosis
Source: Neurol Res Pract. 2026 Oct 21;8(1):87. doi: 10.1186/s42466-026-00544-x (PMC13688721; doi:10.1186/s42466-026-00544-x)
Supplement: Supplementary file 1 [file 42466_2026_544_MOESM1_ESM.docx]

|  |  | Zoster | | | | Pneumococcus | | | | PML | | | | Cryptococcus | | | |
| --- | --- | --- | --- | --- | --- | --- | --- | --- | --- | --- | --- | --- | --- | --- | --- | --- | --- |
|  | all | a | ROR | rmin | rmax | a | ROR | rmin | rmax | a | ROR | rmin | rmax | a | ROR | rmin | rmax |
| Alemtuzumab | 22893 | 631 | 3.54 | 3.26 | 3.85 | 12 | 10.67 | 5.68 | 20.0 | 98 | 1.73 | 1.41 | 2.11 | 13 | 3.75 | 2.13 | 6.61 |
| Cladribin | 17561 | 409 | 2.90 | 2.62 | 3.21 | 1 | 0.95 | 0.13 | 6.85 | 11 | 0.24 | 0.13 | 0.44 | 2 | 0.7 | 0.17 | 2.83 |
| Fingolimod | 101650 | 2047 | 3.12 | 2.97 | 3.28 | 11 | 2.16 | 1.12 | 4.14 | 369 | 1.64 | 1.47 | 1.83 | 120 | 25.58 | 18.25 | 35.8 |
| Fumaric acid | 152761 | 1164 | 1.03 | 0.97 | 1.09 | 6 | 0.71 | 0.31 | 1.66 | 154 | 0.41 | 0.35 | 0.49 | 7 | 0.29 | 0.14 | 0.62 |
| Glatirameracetat | 100415 | 168 | 0.20 | 0.17 | 0.23 | 0 | 0 | 0 | 0.84 | 16 | 0.06 | 0.04 | 0.1 | 0 | 0 | 0 | 0.31 |
| Interferon beta-1a | 215074 | 634 | 0.37 | 0.34 | 0.40 | 6 | 0.51 | 0.22 | 1.18 | 40 | 0.07 | 0.05 | 0.1 | 5 | 0.15 | 0.06 | 0.36 |
| Interferon beta-1b | 68414 | 179 | 0.31 | 0.27 | 0.36 | 0 | 0 | 0 | 1.23 | 17 | 0.1 | 0.06 | 0.16 | 0 | 0 | 0 | 0.46 |
| Natalizumab | 172432 | 1860 | 1.61 | 1.53 | 1.70 | 13 | 1.57 | 0.85 | 2.88 | 1760 | 12.2 | 11.24 | 13.2 | 9 | 0.34 | 0.17 | 0.66 |
| Ocrelizumab | 40070 | 895 | 2.95 | 2.75 | 3.17 | 5 | 2.23 | 0.89 | 5.56 | 50 | 0.49 | 0.37 | 0.65 | 3 | 0.46 | 0.15 | 1.45 |
| Ofatumumab | 26130 | 167 | 0.76 | 0.65 | 0.89 | 3 | 1.98 | 0.62 | 6.31 | 43 | 0.65 | 0.48 | 0.88 | 2 | 0.47 | 0.12 | 1.9 |
| Ozanimod | 6014 | 45 | 0.88 | 0.65 | 1.18 | 0 | 0 | 0 | 14.0 | 3 | 0.19 | 0.06 | 0.6 | 0 | 0 | 0 | 5.19 |
| Peginterferon-beta-1a | 33963 | 74 | 0.25 | 0.2 | 0.32 | 2 | 1.00 | 0.24 | 4.08 | 3 | 0.03 | 0.01 | 0.11 | 0 | 0 | 0 | 0.92 |
| Ponesimod | 456 | 3 | 0.77 | 0.25 | 2.39 | 0 | 0 | 0 | 184 | 0 | 0 | 0 | 4.34 | 0 | 0 | 0 | 68.4 |
| Siponimod | 8579 | 118 | 1.64 | 1.36 | 1.97 | 2 | 3.95 | 0.97 | 16.2 | 9 | 0.41 | 0.21 | 0.78 | 2 | 1.44 | 0.36 | 5.8 |
| Teriflunomid | 50819 | 244 | 0.56 | 0.5 | 0.64 | 1 | 0.32 | 0.04 | 2.33 | 44 | 0.34 | 0.25 | 0.45 | 4 | 0.48 | 0.18 | 1.29 |

Total number of reports for a given drug (“all”) and number of reports for a given infection (“a”), reporting odds ratio (“ROR”) and its 95% confidence interval (“rmin, rmax”).

Correlation of the odds ratio in the study of Oshima et al., (2019) with the RORs for PML related reports as calculated in our study. The power law corresponds to a linear dependency of log(OR) vs. log(ROR) and explains the overwhelming part of the variance (R²=0.96).
